# The FreiBurger: a new optotype for P300-based acuity estimation

**DOI:** 10.1007/s10633-024-09982-x

**Published:** 2024-06-25

**Authors:** Céline Z. Duval, Saskia B. Kaczan, Sven P. Heinrich

**Affiliations:** Eye Center, Medical Center - University of Freiburg, Faculty of Medicine, Freiburg, Germany

**Keywords:** Objective acuity estimation, Low vision, Visual field defect, Optotype

## Abstract

**Purpose:**

Accurate objective assessment of visual acuity is crucial, particularly in cases of suspected malingering, or when the patient’s inability to cooperate makes standard psychophysical acuity tests unreliable. The P300 component of the event-related potentials offers a potential solution and even allows for the use of standard optotypes like the Landolt C. However, low-vision patients with large eccentric visual field defects often struggle to locate the Landolt C gap quickly enough for a P300 to be reliably produced.

**Methods:**

Addressing this challenge, we introduce a novel optotype (the “FreiBurger”) with a critical detail that extends through the optotype’s center. Two experiments, with 16 and 12 participants, respectively, were conducted. In the first, psychophysical acuity estimates were obtained with both the FreiBurger and the Landolt C. In the second, we tested the performance of the FreiBurger, relative to the Landolt C, in eliciting a P300 with undegraded vision, simulated low vision, and in a simulated combination of low vision and visual field constriction.

**Results:**

Comparable psychophysical acuity values (average difference 0.03 logMAR) were obtained for both optotypes. In the P300 recordings, both optotypes produced similar P300 responses under conditions of undegraded vision and low vision. However, with the combination of low vision and constricted visual field, the P300 could only be reliably obtained with the FreiBurger, while the amplitude was drastically reduced with the Landolt C (9.1 µV vs. 2.2 µV; *p* < 0.0005).

**Conclusion:**

The new optotype extends the applicability of P300-based acuity estimation to the frequently encountered combination of low vision and constricted visual field, where Landolt C optotypes fail. Although impairments were simulated in the present study, we assume that the advantages of the new optotype will also manifest in patients with such impairments. We furthermore expect the advantages to apply to time-sensitive psychophysical examinations as well.

## Introduction

Misrepresentation of visual acuity is a significant issue as it can give an individual an undue advantage [1, 2]. Examples include false classification of visual impairments in competitive sports [3] or the attempt to avoid work or military duty [4]. In a broader perspective, suspected non-organic visual dysfunction has been estimated to account for 5% of all cases in a general ophthalmology practice [5, 6]. In the context of welfare support, when reviewing medical testimonials in cases of claimed visual loss, Gräf and Jomaa [7] found that 29% of the individuals were falsely classified as their vision was better than testified.

In addition to certain traditional examination strategies that may provide hints as to the presence of better-than-claimed vision [8], several approaches have been proposed to address the issue more quantitatively. For instance, some studies aimed at making subjective tests more resistant to malingering, or at least to detect inconsistencies through statistical response analyses [9, 10] or a surprise effect [11]. Of particular importance are methods of objective visual acuity estimation that have little reliance on the cooperation of the testee as they measure physiological parameters. In addition to helping identify cases of malingering and non-organic visual loss, the same methods will also be useful for testing young children, people with intellectual disabilities, and patients with suspected psychogenic visual loss [12].

Among the methods for objective estimation of acuity, electrophysiological measurements have proven particularly useful. For example, VEP-based acuity estimation is a well-established method in clinical settings [13]. However, this method has limitations, such as overestimating visual acuity in patients with amblyopia or dysfunction of higher-level visual processing [14, 15].

An alternative approach for measuring visual acuity uses the P300 of the event-related potential and has been developed by Heinrich et al. [16]. It can be used with regular optotype stimuli, including the Landolt C [17], which is the internationally standardized optotype [18]. Compared to VEP-based testing, the combination of P300 and optotypes has the advantage of being more akin to subjective visual acuity measurements [15, 17]. However, the approach has yet to be systematically tested in patients with actual visual impairments; in previous studies low vision has only been simulated by artificially degrading participants’ vision [15, 17].

When using the optotype-based approach of estimating acuity with the P300 in a pilot study on patients with real visual impairments (including central and peripheral visual field loss), we found that patients with very low visual acuity and large eccentric visual field defects reported difficulties in localizing the optotype’s critical detail (the gap of the Landolt C) immediately, as the optotype could not be perceived in its entirety. This is detrimental to its use in an ERP recording that requires stimulus-locked (or, rather, perception-locked) averaging of data [19, 20]. Moreover, testees may make eye and head movements to find the gap. These could induce artifacts in the recorded waveforms, which reduces data quality and may make interpretation difficult [21]. Furthermore, Zhang et al. [22] have shown that the P300 was not measurable in the parietal but only in the frontal areas when participants engaged in a search task rather than a pop out task.

To overcome these challenges, we propose a new optotype dubbed “FreiBurger”.^1^ The FreiBurger only requires viewing the center of the stimulus to recognize the orientation, thus eliminating the need to search for the relevant feature at the outer rim. The optotype is therefore particularly useful for individuals with low visual acuity or large visual field defects, allowing for more accurate and efficient P300 measurements. Most other features, however, are adopted from the Landolt C, including the relative scaling of the whole optotype and its critical detail. We conducted two independent experiments to assess (1) the agreement of psychophysical test outcomes obtained with the FreiBurger and the Landolt C, and (2) the performance of the FreiBurger in a P300 oddball paradigm, in particular in cases of constricted visual field.

## General methods

### Stimuli

Stimuli were presented using a custom software based on PsychoPy (Version 1.85.4) [23]. The program was run on a 21.5-inch iMac A1418, which used an LG 55-inch OLED55B7D monitor. The resolution of the screen was 3.840 × 2.160 pixels, the Weber contrast between background and optotype was nearly 100%, and the background luminance in the range of 100–140 cd/m^2^, depending on the optotype size (the built-in image processing of the screen automatically adjusts the luminance in response to the relative amount of dark and bright parts of the stimulus).This is well within the limits prescribed by the international standard for acuity testing [18].

The experiments were carried out monocularly with participants wearing a trial frame with their habitual correction for the study eye and an occluder for the other eye. In some of the test runs, a degrading filter was used as specified below to simulate reduced visual acuity.

**Fig. 1:**
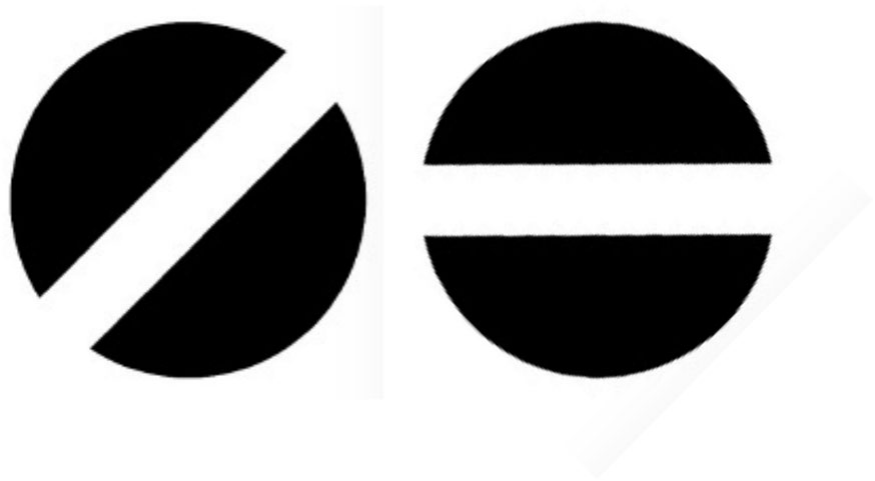
The new “FreiBurger” optotype. The essential feature is that, unlike in the Landolt C, the relevant feature does not have to be searched for at the outer rim, but viewing the center of the stimulus is sufficient to recognize the orientation. The Landolt C’s concept of proportionality between the critical detail and the optotype’s overall size is preserved in the FreiBurger. As with the Landolt C, the width of the gap is one fifth of the total diameter of the optotype

The optotypes used were the standard Landolt C and the newly developed FreiBurger (Fig. 1). The important difference between the two optotypes lies in the critical detail, i.e. the feature that defines the different variants within one optotype design (e.g., the gap that defines the different orientations of an optotype). While the Landolt C’s critical detail is confined to its outer rim, the FreiBurger’s critical detail extends through the center. The optotype’s shape is that of a filled black disk with the gap across the full diameter. The Landolt C’s concept of proportionality between the critical detail and the optotype’s overall size is preserved in the FreiBurger.

### Participants

First Study: 16 participants (10 women, mean age 31) and second study: 12 participants (9 women, mean age 24) were enrolled in the study after providing written informed consent. They were paid €10 the hour. All had normal or corrected to normal vision (0.0 logMAR or better) as confirmed with the Freiburg Acuity and Contrast Test (FrACT) [24], and with no known disorders of the visual or neural system. The study was part of a series of experiments that had been approved by the local review board. It followed the tenets of the Declaration of Helsinki.

## First experiment—assessing visual acuity with the FreiBurger

Although the FreiBurger follows similar design principles as the Landolt C, it is not a priori clear whether their orientation would be equally easy (or difficult) to identify when both have the identical size. In other words, it needs to be tested whether the threshold size in a given individual is the same for both optotypes.

### First experiment—methods

#### Stimuli and procedure

In an acuity test, the relationship between the size of the optotype and the probability of a correct response can be described by a psychometric function [25, 26]. In our first study, we utilized a constant-stimuli approach to estimate psychophysical visual acuity and compared the results from both optotypes.

Each run included 75 optotypes of one style (Landolt C or FreiBurger) in five participant-specific fixed sizes. The gap sizes ranged from 0.2 log arcmin below the individual threshold size to 0.2 log arcmin above the individual threshold size in steps of 0.1 log arcmin (5 different sizes). The individual threshold was determined with the FrACT for the respective vision condition (undegraded or degraded as specified below). All test runs were performed with a screen distance of three meters. The participants were asked to indicate the orientation of the optotype by pressing corresponding buttons on a response keyboard.

We used Landolt C optotypes with eight different orientations corresponding to the four cardinal orientations and the four oblique orientations in between. As the FreiBurger is rotationally symmetric at 180°, it had only four discernable orientations. The first stimulus corresponds to the individual threshold size, all following stimuli were presented in a randomized order with an equal ratio of the different sizes and orientations (15 presentations of each size).

Each participant was tested both with and without degraded vision. Degradation was achieved with a Luminit 1° filter (Luminit Light Shaping Diffusor; Luminit, Torrance, CA, USA). It produces Gaussian blur (cf. [27]) and has been characterized in a previous study [28].

In a 2 × 2 experimental design ([degraded vs. undegraded] × [Landolt C vs. FreiBurger]), each participant completed four different experimental conditions in randomized order. Each condition encompassed two test runs (for test–retest comparison), each lasting 2.5 min on average and including 75 optotypes in 5 different sizes.

#### Analysis

**Fig. 2:**
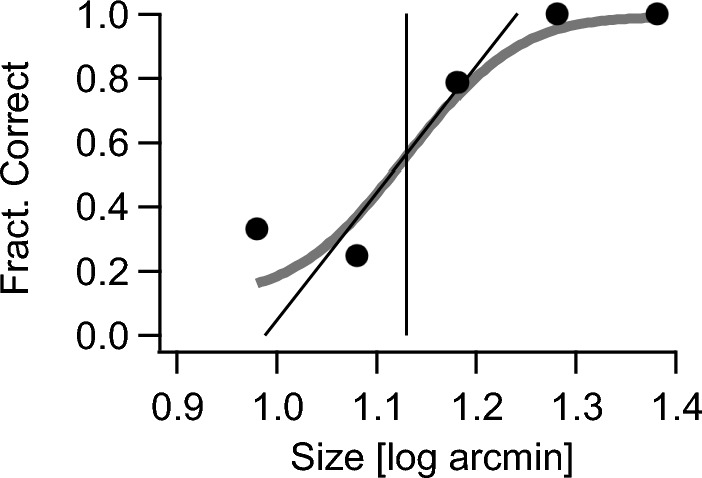
Example of a fitted psychometric function based on the percentage of correct responses to the optotypes of 5 sizes. The fixed optotype sizes were chosen relative to the outcome of a preceding initial acuity measurement. The abscissal value of the inflection point was taken as the acuity value of this measurement, as indicated by the vertical line. The graph also shows the asymptote in the inflection point, which illustrates the slope

Igor Pro 8 (WaveMetrics Inc., Lake Oswego, Oregon, USA) was used to determine the psychometric function by fitting a cumulative gaussian function via a maximum likelihood fit based on all single trials. A lapsus rate of 0.01 was assumed and visual acuity was determined at the inflection point [9] (Fig. 2).

### First experiment—results

**Fig. 3:**
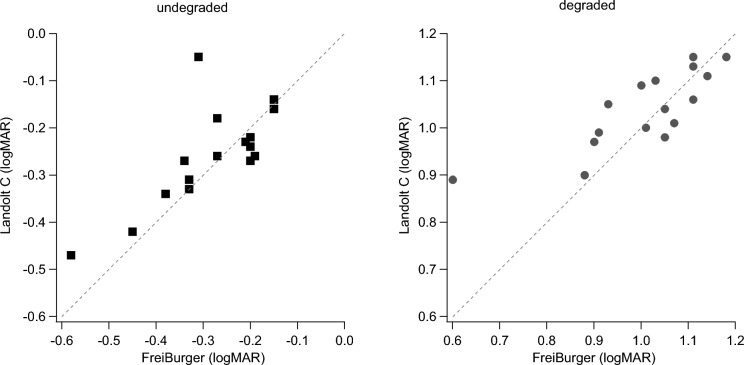
Comparison of the visual acuities obtained with the two optotypes under undegraded (left) and degraded (right) conditions. The FreiBurger yielded slightly better acuity values than the Landolt C

Participants reported no problems with the FreiBurger while performing the test runs. Without degradation, the average logMAR values were − 0.26 (95%-CI: − 0.29 to − 0.23) with the Landolt C and − 0.28 (95%-CI: − 0.32 to − 0.25) with the FreiBurger. With degradation, the respective values were 1.04 (95%-CI: 1.06–1.01) with the Landolt C and 1.01 (95%-CI: 1.05–0.96) with the FreiBurger. The mean of the individuals’ absolute differences between optotype styles were 0.03 both with and without degradation (Fig. 3).

**Fig. 4:**
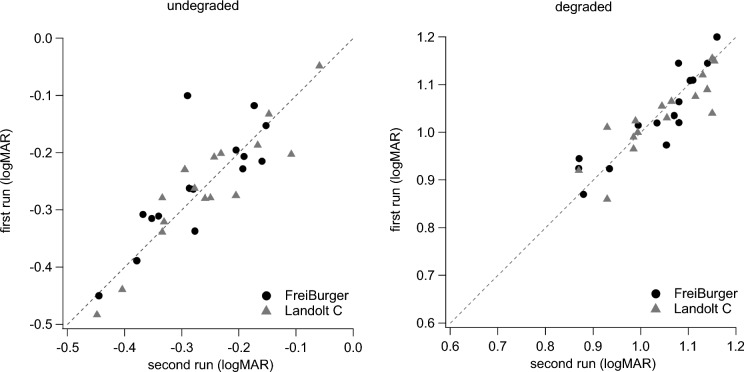
Individual test–retest data obtained with the two optotypes under undegraded (left) and degraded (right) conditions. There is only a little difference between repetitions

The mean of the individuals’ absolute differences between repetitions of the same condition (Fig. 4) without degradation (test–retest reliability) were 0.08 and 0.11 for Landolt C and FreiBurger, respectively. The corresponding values with degradation were 0.06 and 0.1.

### First experiment—discussion

This experiment demonstrates that both optotypes can be used equally to measure visual acuity. The resulting values were nearly identical with a slight trend towards better outcomes (smaller logMAR values) for the FreiBurger. Generally, a small difference is not surprising for two reasons. First, in the case of the FreiBurger, the critical detail extends across the full optotype, which improves the signal-to-noise ratio at sizes near threshold in comparison to the Landolt C where the gap only extends across the stroke width. Second, the effective number of alternative orientations is lower in the case of the FreiBurger. From the perspective of signal detection theory, having fewer alternatives implies having fewer opportunities for false orientations to prevail perceptually over the true orientation at sizes near threshold due to noise [29].

## Second experiment—P300

This experiment aimed at assessing the performance of the FreiBurger as a stimulus for P300 recordings. The P300 is typically recorded to the infrequent stimuli in an oddball sequence [30, 31]. In our study, the infrequent stimuli were optotypes with a gap, while the frequent stimuli were similar in shape except that there was no gap. Optotypes with a gap that were too small in relation the individual’s acuity blended into the sequence of frequent stimuli as the gap could not be resolved. These optotypes were thus not perceived as oddballs and could not elicit a P300.

### Second experiment—methods

#### Stimuli and procedure

**Fig. 5:**
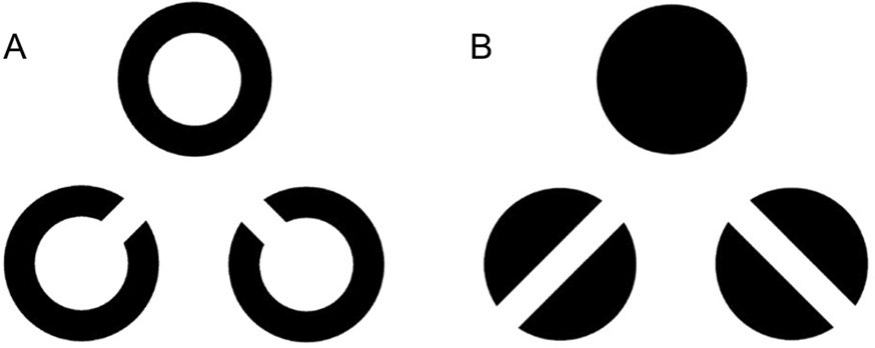
Stimuli used in the P300 paradigm. **A** The Landolt C with two orientations as infrequent stimuli and the closed optotype as frequent stimulus. **B** The FreiBurger with two orientations as infrequent stimuli and the filled ring as frequent stimulus

The infrequent and frequent stimuli were presented in randomized order at a ratio of 1:7 (infrequent:frequent), with infrequent stimuli consisting of either Landolt Cs or FreiBurgers with orientations of ± 45° while the frequent stimuli were closed rings without gap in the Landolt C condition and a completely filled disk in the FreiBurger condition (Fig. 5). Participants were instructed to observe the optotypes and press a button indicating the orientation when an infrequent stimulus (i.e., an optotype with gap) was detected. It is important to note that the response task assigned to participants was not utilized for the actual measurement of visual acuity or the P300 response in this study. Rather, it served to ensure that participants were actively engaged and attending to the stimuli presented on the screen, and to mitigate potential boredom during the experiment.

Given that the primary objective of this study was to compare two optotypes rather than to measure visual acuity, the experimental paradigm was optimized towards this aim while maintaining an acceptable overall duration for the participant. Therefore, only one stimulus size was presented, with a gap size of 2.7 log arcmin that was chosen in consideration of the effects of artificially degraded acuity and constricted visual field as detailed below. Each stimulus was presented for 500 ms, followed by an inter-stimulus interval (ISI) of 500 ms with an empty white screen. Stimulus distance was 80 cm. For each condition approximately 800 stimuli (thus 100 infrequent) were shown to ensure a good signal to noise ratio, resulting in an approximately 15 min duration per condition and a total of 90 min.

To reduce visual acuity, a Luminit 5° filter (Luminit Light Shaping Diffusors; Luminit, Torrance, CA, USA) was used to ensure very large threshold optotype sizes. This is a stronger version of the filter used in Experiment 1. To simulate visual field loss, participants were fitted with a 3.5 mm pinhole in the receptacle of the trial frame furthest from the eye (distance to cornea 19 mm). This pinhole was not used for the typical purpose (to increase the depth of focus), but instead served to produce “tunnel vision”. Although this does not exactly match true visual field loss, it reproduces the essential characteristics for the purpose of the study.

Six experimental conditions were administered in randomized order.

- Undegraded vision, Landolt C
- Undegraded vision, FreiBurger
- Degraded vision, Landolt C
- Degraded vision, FreiBurger
- Degraded vision and constricted visual field, Landolt C
- Degraded vision and constricted visual field, FreiBurger

#### EEG recording and analysis

The electroencephalogram (EEG) was recorded with a 32-channel BrainAmp EEG system with actiCap active electrodes (Brain Products, Gilching, Germany). The signals were filtered with a band-pass range of 0.1–70 Hz and sampled at a rate of 500 Hz. The recordings were referenced to the FCz electrode during data acquisition and re-referenced to the average of TP9 and TP10 (mastoids) for analysis, as a sizable P300-related activation can be expected at FCz. Trials with artifacts such as eye blinks were removed using a ± 100 μV threshold criterion. The recorded trials were pooled based on stimulus type and condition, and low-pass filtered at 25 Hz. ERPs were extracted through standard averaging after trial-by-trial baseline correction using the − 100 to + 50 ms interval around stimulus onset as a baseline reference. Analysis primarily focused on the Pz electrode, which typically shows the highest P300 amplitude [19, 32].

In order to isolate the oddball-related components of the ERP, difference curves were computed by subtracting the response to frequent stimuli from the response to infrequent stimuli. This removes any activity that is common to both responses. For group-level comparisons of the amplitudes, we used a Wilcoxon signed rank test.

The statistical significance of the ERPs to individual stimulus categories and visual degradation conditions was determined for each participant using a bootstrap test with 5000 samples that compared the mean ERP amplitude within an interval of 200–600 ms after the stimulus between infrequent and frequent stimuli. All data analyses were performed using Igor Pro 8 (WaveMetrics Inc., Lake Oswego, Oregon, USA).

### Second experiment—results

#### Group level

**Fig. 6:**
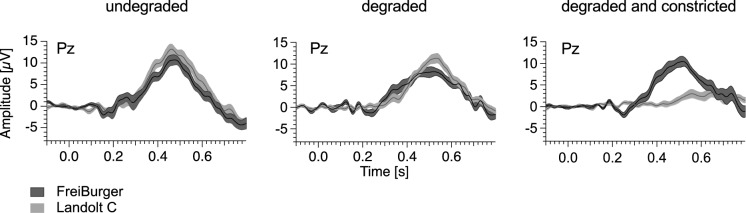
Grand mean difference ERP traces from all visual acuity categories (infrequent minus frequent, revealing any P300) measured at Pz. The dark grey traces represent results obtained with the FreiBurger, the light grey traces represent results obtained with the Landolt C. With degradation and constricted visual field, the FreiBurger performed much more reliable in eliciting a P300

Figure 6 provides an overview of the grand mean difference curves at Pz revealing the presence (or absence) of a P300 for each optotype style, separated by condition. In the conditions without visual degradation (Landolt C, median x̃ = 9.7 μV IQR = 7.6 μV; FreiBurger, x̃ = 6.2 μV IQR = 3.3 μV), as well as in the conditions with visual degradation (Landolt C, x̃ = 8.4 μV IQR = 3.6 μV; FreiBurger x̃ = 7.0 μV IQR = 3.5 μV), the difference in P300 peak amplitude between both optotypes are small. However, in the condition with visual degradation and constricted visual field, P300 has drastically reduced amplitude with the Landolt C compared to the FreiBurger x̃ = 1.6 μV IQR = 2.1 μV versus x̃ = 9.8 μV IQR = 4.1 μV and is furthermore considerably delayed.

Although the behavioral task during the P300 experiment only served to keep the participants attentive, we nevertheless performed some checks in order to spot potential problems that might have occurred. With undegraded and degraded vision (without simulated visual field constriction), participants detected 90–100% of the target stimuli with a single exception (87%), irrespective of when the respective condition occurred during the course of the experimental session. Detection rates were somewhat more variable with constricted visual field when the FreiBurger was used (70–98%, with one exception of 51%) and a slight effect of randomization sequence. When the Landolt C was used with visual field constriction, detection rates were 0–56% with some effect of randomization sequence (worse performance when the condition occurred later in the session). Because of the small number of participants with a similar randomization sequence, we refrained from a detailed quantitative analysis.

#### Single-participant level

**Fig. 7:**
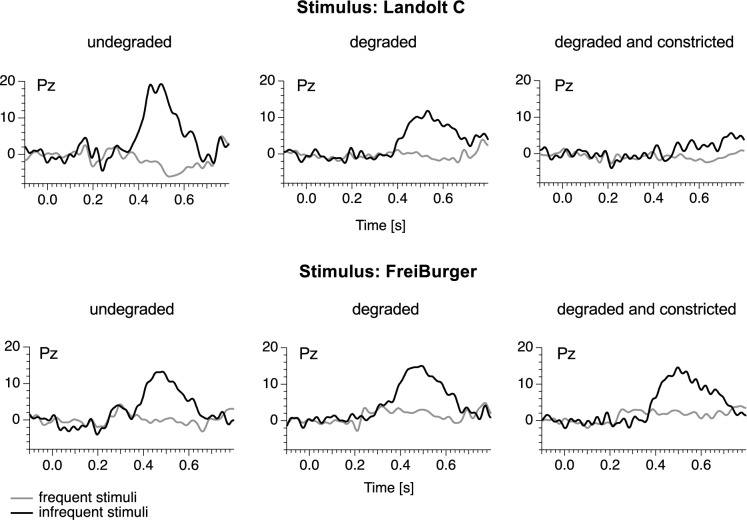
Individual original data for one single participant. In the case of a constricted visual field, no P300 was recorded with the Landolt C, while a clear P300 was obtained with the FreiBurger

The original ERP curves of one individual are presented in Fig. 7. For each condition, we computed the average response data to the frequent stimulus separately from the response to the infrequent stimuli.

**Fig. 8:**
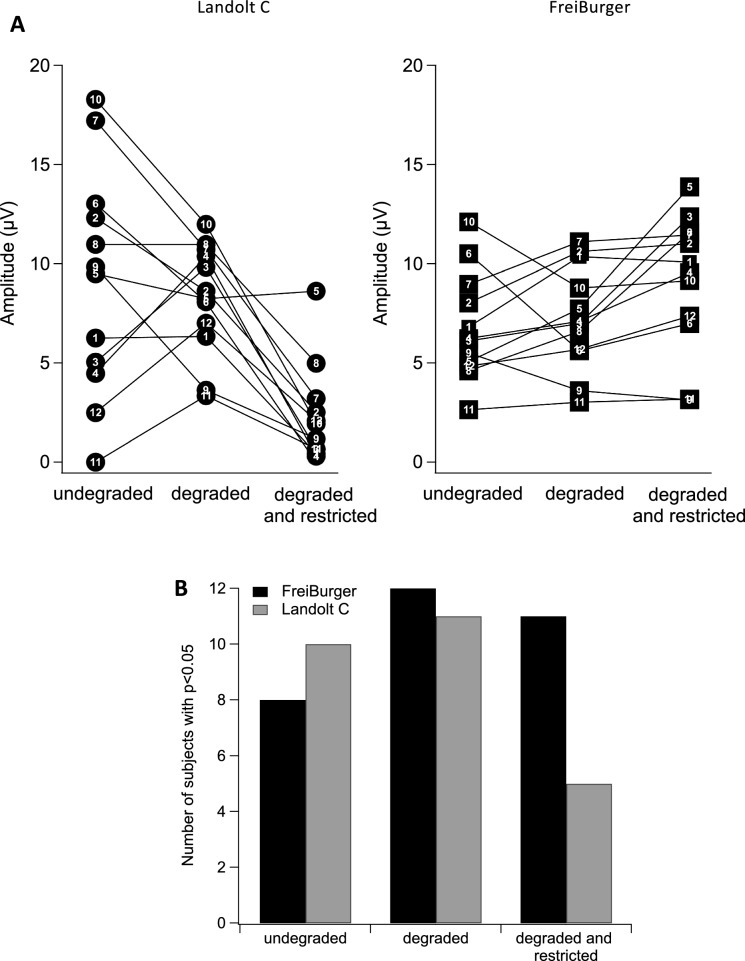
**A** P300 amplitudes for individual participants at Pz as a function of viewing condition. On the left side the results using the Landolt C, on the right side the FreiBurger. Each number identifies an individual participant. **B** Number of participants (of a total of 12) with a significant P300 at Pz (*p* < 0.05, bootstrap test), separately for the FreiBurger (black) and the Landolt C (gray). With the FreiBurger, almost all participants produced a significant response when the visual field was constricted, while this was only the case in 5 participants with the Landolt C

To evaluate the findings at the single-participant level, we applied a bootstrap test to the ERP curves [33], focusing on the interval from 200–600 ms after stimulus onset. The analysis aimed to assess the significance of the oddball responses relative to the frequent stimuli for each participant individually (Fig. 8). Without degradation, a clear majority of participants produced a significant P300. Interestingly, these numbers were even higher with degradation. When, in addition to acuity degradation, the visual field was constricted, the number of individuals with a significant response dropped substantially with the Landolt C, but not so with the FreiBurger.

Peak time analysis of the P300 to infrequent stimuli showed only minor differences between most conditions (undegraded, FreiBurger: median x̃ = 0.47 s IQR = 0.05 s; undegraded, Landolt C: x̃ = 0.42 s IQR = 0.08 s; degraded, FreiBurger: x̃ = 0.46 s IQR = 0.09 s; degraded, Landolt C: x̃ = 0.51 s IQR = 0.04 s; degraded and constricted field, FreiBurger: x̃ = 0.52 s IQR = 0.05 s). We refrained from analyzing peak times in the condition with Landolt C stimuli when degradation and constriction were combined, as these times appeared noise-dominated in several participants in the absence of a sizable P300.

### Second experiment—discussion

With both optotypes, including the FreiBurger, reliable P300 responses could generally be recorded. However, with visual degradation and constricted peripheral visual field, the oddball response to Landolt C stimuli was weak and delayed, while the response to the FreiBurger was virtually unaffected. The aim of making optotype-based acuity estimation with the P300 more robust in cases of visual field defects has thus been achieved.

One might wonder whether the weak positivity obtained with Landolt Cs in the degraded condition with constricted visual field would have been detected more reliably if the analysis interval had been shifted in time accordingly. Testing this for the 500–800 ms range, we found statistical significance only in one additional participant. We assume that a mixture of several effects causes the amplitude reduction, namely a large variability in P300 timing, resulting from the variable delay in the recognition of the eccentric Landolt C gap, and trials being included in the ERP average in which the gap went completely unnoticed and thus could not elicit any P300. It should be noted, though, that the experiment did not directly assess the time course of optotype recognition.

The spot checks on the behavioral data suggest that the issue of missing Landolt C target stimuli when the visual field is constricted might be aggravated by fatigue, as there was some evidence of reduced performance when the respective condition occurred late in the sequence of randomized conditions. The FreiBurger appeared to be less affected. These effects need to be interpreted cautiously, though, because of the low number of participants with the same sequence positions for the respective conditions.

## General discussion

In the present study, we investigated the efficacy of the newly developed FreiBurger optotype for objective visual acuity measurements. Our psychophysical findings suggest that the FreiBurger is a valid alternative for visual acuity measurement and in the EEG experiment the novel optotype shows a clear advantage over the Landolt C in a P300 oddball paradigm at low visual acuity with constricted visual field.

At first glance, the issue dealt with in here may seem like a niche problem. However, the FreiBurger substantially improves the applicability of P300-based acuity testing in the respective use scenarios. In particular, the proposed optotype helps to not confound low visual acuity and visual field defects as outcome determinants in P300-based acuity estimation with regular Landolt Cs or other optotypes that would have an eccentric critical detail. Another field of application would be psychophysical tests that involve the measurement of response time. Such a test would not produce a meaningful result if most of the response time is required for actively searching the critical detail by moving the eyes or the head. An example of such a time-critical test has been presented by Graf and Roesen [11], who have used response times to identify cases of malingering and misrepresentation.

The optotype proposed here is somewhat reminiscent of the optotype described by Pishnamaz and Ostadimoghaddam [34]. However, their optotype is more akin to a grating confined to a circular aperture. It was apparently designed with a different goal, namely measuring astigmatism in addition to acuity, while the present study aims at extending the critical detail of the Landolt C to the center of the optotype. Pishnamaz and Ostadimoghaddam [34] did not evaluate the performance of their proposed optotype. Although the design aims and characteristics of the optotypes differ, both studies highlight that specialized optotypes have advantages over standard optotypes in certain applications. The FreiBurger also shares some similarities with the “double bar” stimulus by Benda [35], which consists of two black bars separated by a white stripe, with the whole arrangement confined to an octogonal aperture. With that stimulus, average acuity values were worse than with the Landolt C (decimal VA 1.06 vs. 1.34, corresponding to logMAR − 0.025 vs. − 0.13). This is opposite and different in magnitude to what we found with the FreiBurger.

The P300 with the FreiBurger was largest when vision was both degraded and constricted. Possibly, the percept of the white bar of the infrequent FreiBurgers was experienced as particularly salient when occurring within the constricted visual field, causing the P300 to be enhanced [36]. We cannot exclude that, in addition to the shape percept of the white bar, the luminance effect within the constriction aperture, which resulted from the presence of the bar, contributed to the salience of the infrequent stimuli.

Although the published recommendations [18, 37] only require a comparison to be performed in individuals with good acuity, we evaluated the new optotype also with degraded vision and found good agreement. Nevertheless, we cannot predict with absolute certainty that this will hold for all types of visual impairment. This is, of course, a general problem pertaining to all optotype designs, and a discrepancy does not necessarily imply that the results obtained with one design are more correct than those obtained with the other design.

The P300 experiment of the present study did not include a condition with central visual field loss. However, it seems safe to assume that the FreiBurger would not perform worse than the Landolt C in such a case, as both optotypes have their critical detail extend to the outer rim of the optotype shape. In patients with central visual field loss who use an eccentric preferred retinal locus for fixation [38] the FreiBurger might even have an advantage.

In conclusion, using the FreiBurger optotype in an oddball paradigm with P300 ERP is a promising approach for objectively measuring visual acuity in individuals with very low vision and extensive visual field defects.

## Data Availability

10.6084/m9.figshare.24135315.v1

## Footnotes

1 The Name “FreiBurger” was selected because the optotype visually resembles the two halves of a hamburger bun and has its origins in the city of Freiburg.

## Declarations

### Statement on the welfare of animals

This article does not contain any studies with animals other than *homo sapiens* performed by any of the authors.
